# Posterior Shoulder Stability Can Be Restored by Posterior Acromial Bone Grafting (Scapinelli) in a Cadaveric Biomechanical Model With Normal Glenoid Anatomy

**DOI:** 10.1177/03635465251362854

**Published:** 2025-08-18

**Authors:** Bettina Hochreiter, Nhi Nguyen, Anna-Katharina Calek, Bastian Sigrist, David C. Ackland, Lukas Ernstbrunner, Eugene T. Ek, Christian Gerber

**Affiliations:** † Melbourne Orthopaedic Group, Melbourne, Victoria, Australia; ‡ Department of Orthopedics, Balgrist University Hospital, University of Zurich, Zurich, Switzerland; § Department of Biomedical Engineering, University of Melbourne, Melbourne, Victoria, Australia; ‖ Research in Orthopedic Computer Science Group, Balgrist University Hospital, University of Zurich, Zurich, Switzerland; ¶ Department of Orthopaedic Surgery, Box Hill Hospital, Box Hill, Victoria, Australia; # Department of Surgery, Monash Medical Centre, Monash University, Melbourne, Victoria, Australia; ** Orthopaedic Research Center, Balgrist Campus, Zurich, Switzerland; Investigation performed at the Department of Biomedical Engineering, University of Melbourne, Melbourne, Victoria, Australia

**Keywords:** shoulder, instability, posterior instability, glenohumeral instability, acromion, acromial morphology, posterior acromial bone graft, posterior acromial bone block, Scapinelli

## Abstract

**Background:**

A high and flat acromion seems to be a risk factor for posterior shoulder instability. Biomechanically, the surgical correction of acromial malalignment can restore glenohumeral joint stability.

**Purpose/Hypothesis:**

The purpose was to assess (1) the stabilizing effect of a posterior acromial bone graft (PABG) in moderate and severe acromial malalignment (high and flat) and (2) contact patterns under posterior humeral head displacement. It was hypothesized that a PABG would significantly (1) increase resistance to posterior humeral head displacement, (2) restore stability, and (3) increase acromiohumeral contact pressure.

**Study Design:**

Controlled laboratory study.

**Methods:**

A total of 8 fresh-frozen human cadaveric shoulders, with normal glenoid anatomy, were examined in a shoulder simulator in the load and shift and jerk test positions. Each specimen underwent 5 testing conditions using 3-dimensional printed cutting and reduction guides, with the joint left intact for each condition: (1) severe acromial malalignment, (2) severe acromial malalignment + PABG, (3) moderate acromial malalignment, (4) moderate acromial malalignment + PABG, and (5) corrected acromial alignment. The humeral head was translated posteriorly until reaching either a peak force of 150 N or a maximum posterior displacement of 50% of the glenoid width. Force, displacement, and acromiohumeral contact pressure were recorded.

**Results:**

At 30° of flexion, the force needed to displace the humeral head 50% increased by 659% when a PABG was added to a moderately malaligned acromion and by 1249% when a PABG was added to a severely malaligned acromion. At 60° of flexion, it increased by 293% and 348%, respectively. This stabilizing effect increased progressively with increasing displacement (*P* < .05 for all comparisons after ≥5% of displacement). Compared with acromial correction, a PABG allowed comparable posterior displacement but required different amounts of force, depending on the scenario. At 30° of flexion after 30% of displacement, a PABG provided significantly greater stability (*P* < .05 for all comparisons). Mean contact pressure was significantly reduced on the rotator cuff and significantly increased on the acromial undersurface in moderate and severe acromial malalignment, whereas a PABG restored acromiohumeral contact pressure comparable with corrective osteotomy, particularly at 30° of flexion.

**Conclusion:**

The study provides quantitative evidence showing that a PABG significantly enhanced resistance to displacement and compensated for deficient posterolateral acromial coverage by extending the natural mechanical buttress.

**Clinical Relevance:**

Experimentally, a PABG provided comparable or superior stability to that after surgical acromial reorientation while representing a technically simpler and potentially less invasive approach.

Posterior shoulder instability is increasingly recognized as a significant cause of shoulder pain and dysfunction.^18,26,31,39,40^ Clinical and biomechanical evidence supports that factors such as posterior capsulolabral tears, glenoid retroversion, and posterior glenoid bone loss contribute to decreased resistance against posterior humeral head displacement.^4,6,7,20,21,24,25^ If nonoperative measures fail, common surgical options include capsulolabral repair, glenoid osteotomy (± J-graft), and posterior glenoid bone augmentation or combinations thereof. However, these procedures have shown failure rates of up to 35% for capsulolabral repair,^3,8,13,28,37^ up to 73% for open posterior bone block procedures,^5,33^ and up to 33% for glenoid osteotomy^12,16,34^ at long-term follow-up.

A high and flat acromion, with poor posterior coverage of the humeral head, has recently emerged as a risk factor for static and dynamic posterior instability^2,22,23^ as well as posterior glenoid bone loss.^19^ Biomechanically, it has been shown that the acromion acts as a mechanical buttress to posterior humeral head displacement and that the surgical correction of acromial malalignment can not only effectively restore but also increase glenohumeral joint stability.^14,15^

In 1973, Scapinelli^30^ proposed bone grafting of the posterolateral acromion, with an autograft taken from the scapular spine, in patients with both atraumatic (n = 8) and traumatic (n = 2) posterior instability, with the intent to put pressure on the infraspinatus. Overall, 7 cases were treated without capsular plication and obtained the same excellent clinical results as the 3 cases with additional capsular plication. He reported no recurrence of instability in 10 patients at a mean follow-up of 9.6 years.^29^

It was therefore the purpose of this study to assess (1) the stabilizing effect of a posterior acromial bone graft (PABG) in moderate and severe acromial malalignment (high and flat) and (2) contact patterns under posterior humeral head displacement. The hypotheses were that a PABG would significantly (1) increase resistance to posterior humeral head displacement, (2) restore stability, and (3) increase acromiohumeral contact pressure.

## Methods

**Figure 1.**
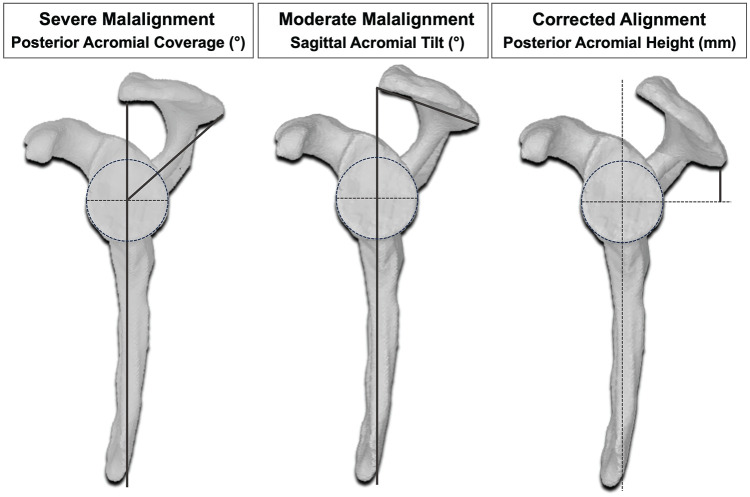
Illustration of the anatomic differences between 3 surgically achieved conditions (No. 1, 3, and 5)^15^ as well as acromial alignment measurements on segmented 3-dimensional models. The vertical dark gray lines correspond to the scapular plane. Bold lines correspond to specific measurements.

Ethical approval was granted for this controlled laboratory study. A total of 8 fresh-frozen cadaveric shoulders were procured, all free from glenohumeral arthritis, rotator cuff tears, and previous injuries or surgery, as confirmed by computed tomography (CT) and visual inspection, from the University of Melbourne's Body Donor Program. These specimens had a mean age of 77.8 years, ranging from 68 to 94 years, and included 4 male and 4 female donors.^15^ CT was also used to assess several parameters: native glenoid width, glenoid retroversion, posterior acromial coverage (PAC), sagittal acromial tilt (SAT), and posterior acromial height (PAH) (Figure 1).

### Testing Conditions and Planning of Acromial Malalignment

The mean native glenoid width was 25.4 mm (range, 21-31 mm), glenoid retroversion was 3.1° (range, 1.3°-5.8°), SAT was 58.6° (range, 41.1°-72.6°), PAC was 63.6° (range, 54.4°-77.6°), and PAH was 18.9 mm (range, 8.5-24.5 mm).

**Figure 2.**
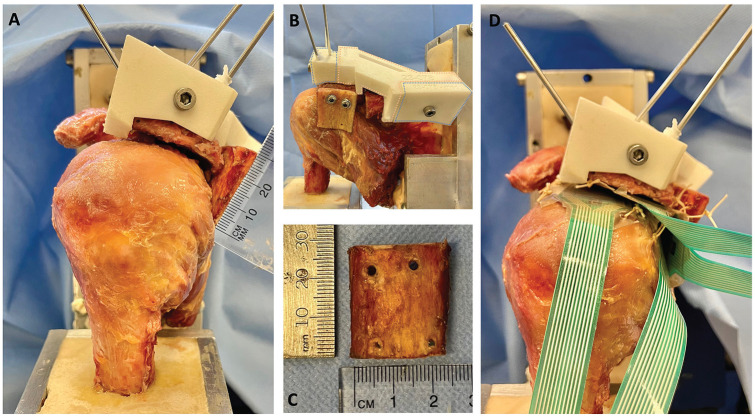
A posterior acromial bone graft (PABG). (A-C) The lateral aspect of the graft was positioned in line with the lateral border of a severely malaligned acromion and oriented perpendicularly to the plane of the acromion. (B) The 3 different parts of the 3-dimensional printed reduction guide: an anterolateral part (blue) and a posteromedial part (blue), which were consistent throughout scenarios, and an interchangeable middle part (pink). (C) The PABG measuring approximately 2.5 × 2.5 cm. (D) Position of the 4 Tekscan sensors and fixation technique.

The categorization of acromial malalignment as moderate or severe was derived from a previous study that utilized 3-dimensional (3D) surface models of segmented CT scans to define acromial anatomy in patients with posterior instability.^2^ Moderate acromial malalignment corresponded to average values observed in patients with dynamic posterior instability: SAT of 59°, PAC of 57°, and PAH of 20 mm.^2^ Severe malalignment was defined as the average values plus one standard deviation (SD), resulting in a SAT of 69°, PAC of 47°, and PAH of 26 mm. Conversely, for corrected acromial alignment, the average values minus one SD from healthy controls without shoulder instability were used: SAT of 48°, PAC of 70°, and PAH of 11 mm (Figures 1 and 2).

Each of the specimens underwent the following testing conditions in chronological order, with the glenohumeral joint left intact for each condition:

1. Severe acromial malalignment (SAT of 69°, PAC of 47°, PAH of 26 mm)^15^
2. Severe acromial malalignment + PABG
3. Moderate acromial malalignment (SAT of 59°, PAC of 57°, PAH of 20 mm)^15^
4. Moderate acromial malalignment + PABG
5. Corrected acromial alignment (SAT of 48°, PAC of 70°, PAH of 11 mm)^15^

In a previous study using the identical experimental setup and specimens, we compared data from native specimens to conditions 1, 3, and 5^15^ to demonstrate differences in posterior humeral head displacement and acromiohumeral contact pressure between acromial malalignment and realignment. Consequently, we did not repeat this comparison in the present study.

Semiautomatic segmentation was conducted using 3D Slicer (Version 5.6.0; https://www.slicer.org/), and the segmented CT scans were then imported into CASPA software (Computer Assisted Surgery Planning Application; Balgrist CARD). This software was used for measuring all the abovementioned values and for planning acromial osteotomy and its orientation for the scenarios described (B.S.). Vertical acromial osteotomy was simulated, positioned 10 mm medial to the glenoid. The acromion of each specimen was adjusted to represent moderate and severe malalignment, as well as corrected alignment, using the predefined values of SAT, PAC, and PAH for each condition. For each specimen, personalized cutting and reduction guides were designed using CASPA and fabricated with a 3D printer (selective laser sintering; FORMIGA P 110 Velocis [EOS]) using fine polyamide 12 (nylon/PA 2200).

### Specimen Preparation and Setup

All specimens were thawed at room temperature for 24 hours before testing. To prevent dehydration, we kept the specimens moist with phosphate-buffered saline during preparation and testing. A fellowship-trained shoulder surgeon (B.H.) performed all surgical procedures. The scapula and humerus were dissected free from surrounding soft tissue, including the clavicle. The supraspinatus and infraspinatus muscles were elevated to expose the scapular spine. The rotator cuff muscles and their tendinous attachments were preserved as well as the capsuloligamentous complex of the glenohumeral joint. The rotator interval was vented. The humeral shafts were osteotomized 15 cm distal to the highest point of the humeral head. Both the scapulae and humeri were secured in custom fixtures using polymethyl methacrylate. Before potting, the exposed bony ends were stabilized with multiple bicortical screws to enhance rotational stability at the bone-potting interface. The scapula was set within a box-shaped fixture, aligning the scapular plane parallel to the lateral borders of the fixture and positioning the glenoid face parallel to the fixture's floor, achieving 0° of glenoid version.^6,15,38^ This precise alignment was reliably achieved by placing a 3D printed cutting guide on the scapular spine.^15^ K-wires, indicating the scapular and glenoid planes, were inserted through predefined holes in the guide. This same guide was also used to osteotomize each scapular spine in the predefined planes with a saw blade.

### Creation of Acromial Malalignment and Correction

After performing osteotomy, reduction guides were positioned on the specimen (Figure 2, A and B). These guides included an anterolateral part and a posteromedial part, consistent across all scenarios. The third middle part of the guides was interchangeable, varying with each surgical condition. It comprised (1) a moderate malalignment block, (2) a severe malalignment block, and (3) a corrected alignment block (Figure 2B). The reduction guides were employed to maintain the lateral acromial fragment in the predetermined position during testing, ensuring rigid fixation of the acromion after osteotomy. The guide blocks were engineered to attach securely to specific points on the bone, interlock with each other, and be compressed using a screw and nut for stable fixation. Additional stabilization was achieved using K-wires (Figure 2, A and B).

### Posterior Acromial Bone Graft (Scapinelli)

A 2.5 × 2.5–cm bone block (Figure 2C) was harvested from the resected humeral shaft of the largest male specimen using an oscillating saw. To minimize variability in bone quality, graft size, and curvature, the same bone graft was used across all specimens and testing conditions. The acromion's posterolateral edge was identified and cleared of any soft tissue. The lateral aspect of the graft was positioned in line with the lateral border of the acromion and oriented perpendicularly to the plane of the acromion (Figure 2A). It was then temporarily fixed with 1.6-mm K-wires. Using a cannulated drill, 2 holes were drilled for 4-mm partially threaded screws, which were then fixed in a bicortical fashion through the bone block to the anterior aspect of the acromion with a washer (Figure 2B).

### Stability Testing

**Figure 3.**
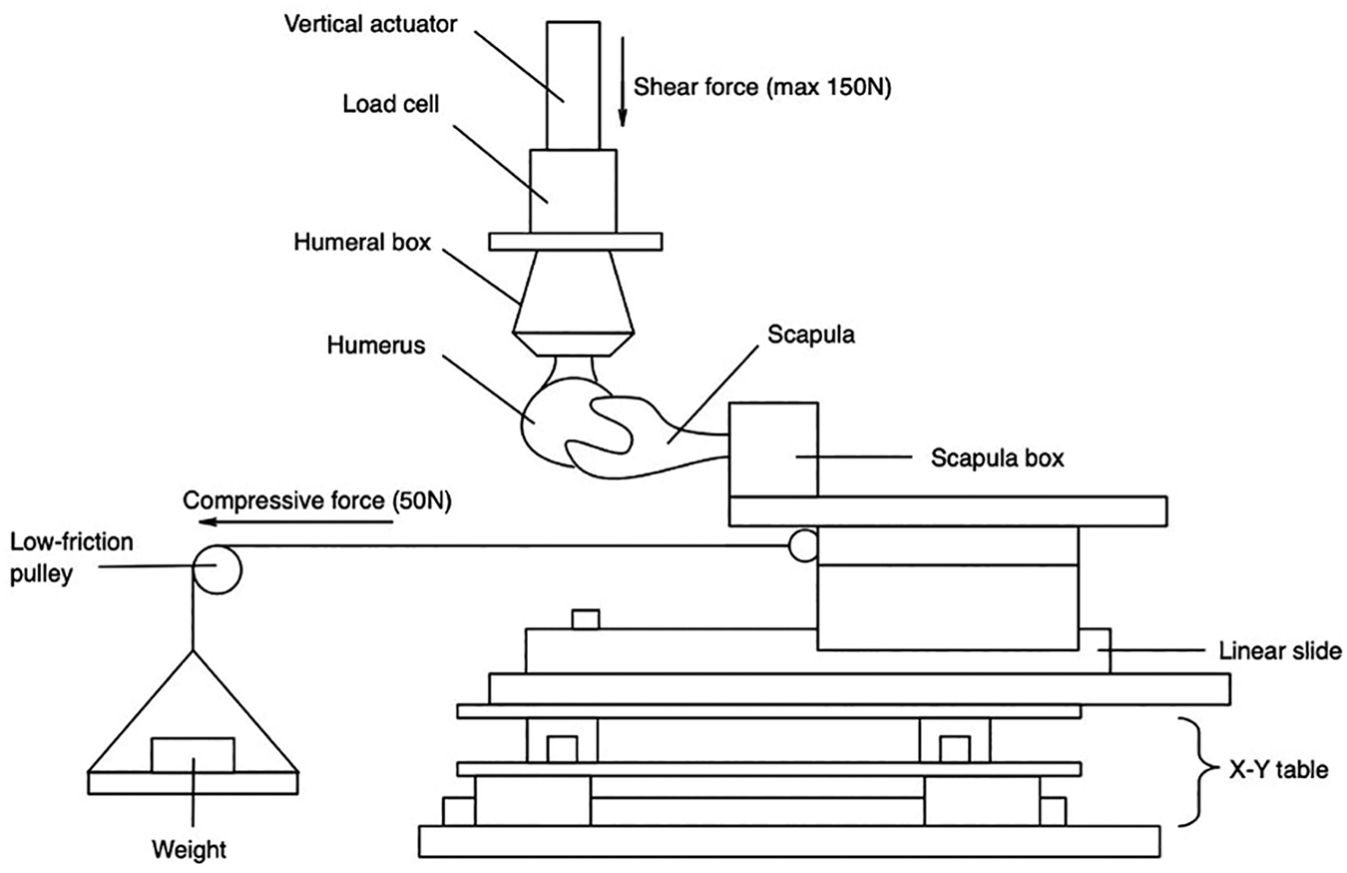
Illustration of the custom-made shoulder simulator. Arrows point in the direction of posterior directed force (maximum, 150 N) and compressive force (50 N) generated by a static weight via a lever arm. Anteroposterior displacement was generated by the vertical actuator. The linear slide allowed for mediolateral translation, whereas the X-Y table allowed for superoinferior translation. Adapted from Figure 4 in a study by Hochreiter et al.^15^

The specimens were mounted onto a custom-built shoulder testing system (Figure 3). The scapular box was secured to a horizontal linear bearing translator and lever arm system, situated on top of 2 translation plates that enabled mediolateral and superoinferior movements. The humerus was mounted to the upper crosshead of the materials testing machine (8874; Instron) equipped with a 6-axis force-torque sensor (K6D68; ME Systeme). Testing was conducted in 2 distinct positions to replicate (1) the load and shift test and (2) the jerk test. The load and shift test, defined as neutral adduction, 30° of glenohumeral flexion, and 45° of internal rotation, reproduces a position in which the glenohumeral articulation provides most of the stability rather than the ligamentous and tendinous structures.^24,25^ The jerk test, traditionally associated with provoking posterior instability, was performed with the specimens in neutral adduction, 60° of glenohumeral flexion, and for simplicity in the setup, 45° of internal rotation instead of the usual 60° described in typical protocols.^17,36^ A compressive load of 50 N was applied perpendicularly to the glenoid using the horizontal linear bearing translator. This setup allowed the humeral head to preliminarily self-center within the glenoid concavity.^6,35,38^ Definitive positioning within the center of the glenoid concavity was achieved when the anteroposterior force measured by the Instron machine balanced to 0 N.^24^ This state was termed the reference-neutral position and served as the baseline for each testing condition.^6,24,27^ After defining the starting position, superoinferior translation of the scapula was restricted by blocking the respective translation plate. From this neutral position, the humeral head was translated posteriorly at a consistent rate of 10 mm/min.^9^

Testing was halted upon reaching 1 of 2 predefined endpoints: either a peak force of 150 N or a maximum posterior displacement equivalent to 50% of the glenoid width. These limits were set based on preliminary experiments to avoid dislocations and capsulolabral damage. Force (N) and displacement (mm) were continuously recorded throughout testing.

### Acromiohumeral Contact Patterns and Mean Contact Pressure

During testing, contact patterns and mean contact pressure along the rotator cuff as well as the undersurface of the acromion and the PABG were simultaneously recorded using dynamic pressure pads. These pads were 0.102 mm thick and incorporated a 14 × 14–mm sensel matrix with a resolution density of 62 sensels/cm^2^ (Sensor 6900; Tekscan). Each sensor was precalibrated following the manufacturer's guidelines. The sensors were strategically placed: one on the undersurface of the acromion, aligned with the posterolateral edge; another just superior to the junction of the supraspinatus and infraspinatus on top of the supraspinatus tendon, 3 cm medial to its insertion; a third just inferior to the junction on top of the infraspinatus tendon; and a fourth on the undersurface of the PABG (Figure 2D). The positions of the sensors were marked and secured with sutures to maintain accuracy throughout the testing process.^6,11,27^ The same force-controlled protocol, applying a posteriorly directed force of 150 N, was employed to analyze mean acromiohumeral contact pressure (kPa).

To ensure data normalization relative to specimen size, particularly to account for variations in glenoid width, displacement (mm) was reported as a percentage of each specimen's individual glenoid width. Testing was conducted in both positions outlined previously, across each of the 4 described testing scenarios. Each condition was tested 3 times, and average values were subsequently used for analysis. Upon completing all testing scenarios, the rotator cuff of each specimen was reflected, and the capsule was incised at its lateral insertion to inspect the posterior labrum and assess its integrity. Notably, no macroscopic damage to the labrum or the capsuloligamentous structures was observed in any of the specimens.

### Statistical Analysis

Using G*Power (Version 3.1.9.6), an a priori power analysis was performed for estimating the sample size, based on a pilot study with 2 specimens. The pilot study recorded an effect size of 2.39, categorized as small according to the Cohen *d*. To meet a significance of alpha of .05 and a power of 0.80, the calculated minimum sample size required was 8 for conducting 2-way repeated-measures analysis of variance (ANOVA).

Data normality was verified using the Shapiro-Wilk test. Force-displacement data were assessed using 2-way repeated-measures ANOVA, while acromiohumeral contact patterns were evaluated via 1-way repeated-measures ANOVA. Because of the violation of sphericity, the Greenhouse-Geisser correction was applied to adjust the degrees of freedom for the *F* test. Post hoc comparisons were made using the Tukey test to control for multiple comparisons.

For each testing condition in every specimen, a polynomial curve was fitted to all measurements up to the maximum force of 150 N. In instances in which posterior displacement had to be halted before reaching 50% of the glenoid width, to prevent exceeding 150 N and thus avoid potential soft tissue damage or acromion fractures, the dataset became inhomogeneous. To address the missing data points and allow for valid statistical comparisons between conditions that reached the maximum force and those that reached maximum displacement, curve extrapolation was employed to complete the dataset.

Absolute values (mean ± SD) for all measurements, along with mean differences and 95% confidence intervals for the comparisons, are presented in Appendix Tables A1 to A4 (available in the online version of this article). All statistical analyses were carried out with a significance level set at .05 using Prism software (Version 10.0.3; GraphPad).

## Results

### Stability Testing

**Figure 4.**
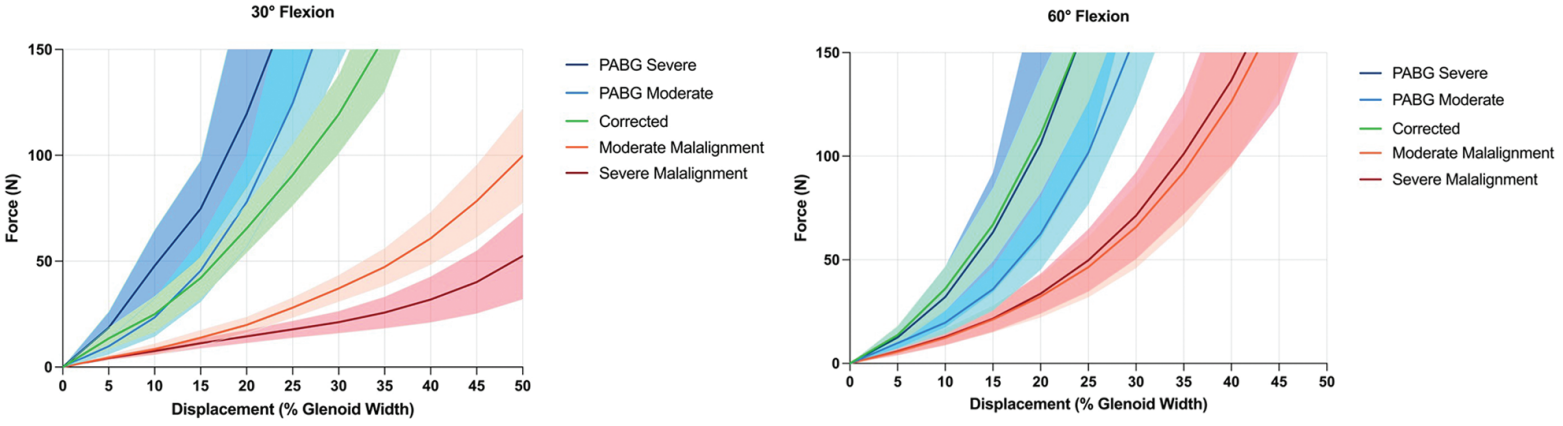
Force-displacement curves for all testing conditions at 30° and 60° of glenohumeral flexion. At 30°, the acromial correction and posterior acromial bone graft (PABG) conditions reached the force limit of 150 N between approximately 23% and 34% of displacement, while the 2 malaligned conditions reached 50% of displacement at varying lower levels of force. At 60°, all conditions reached the force limit of 150 N.

**Figure 5. fig5-03635465251362854:**
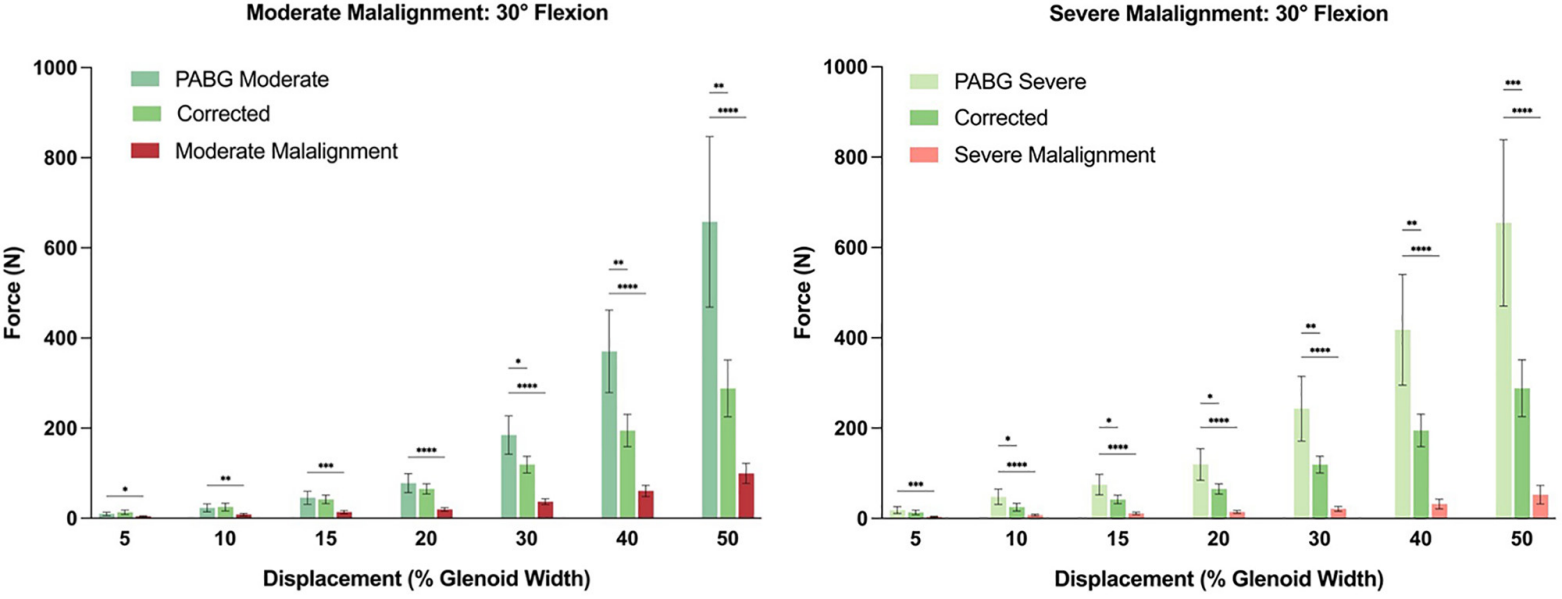
Statistical comparison of the force (N) that was reached, and would have been reached, per condition at different magnitudes of displacement (% glenoid width) at 30° of glenohumeral flexion. *P < .05; **P < .01; ***P < .001; ****P < .0001.

At both 30° and 60° of flexion, the addition of a PABG to either a moderately or severely malaligned acromion significantly increased stability after only ≥5% of posterior humeral head displacement. This stabilizing effect increased progressively with increasing displacement (Figures 4 [Fig fig5-03635465251362854]-6 and Appendix Tables A1 and A2).

**Figure 6.**
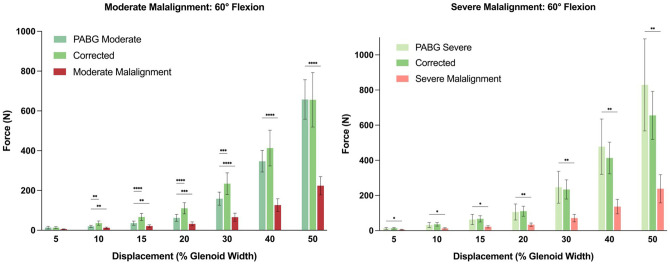
Statistical comparison of the force (N) that was reached, and would have been reached, per condition at different magnitudes of displacement (% glenoid width) at 60° of glenohumeral flexion. *P < .05; **P < .01; ***P < .001; ****P < .0001.

Compared with acromial correction, the PABG (in both moderately and severely malaligned conditions) demonstrated comparable stabilizing effects at 30° of flexion up to 30% of displacement. Beyond 30% of displacement, the PABG allowed significantly less displacement. At 60° of flexion, with moderate malalignment, the PABG provided significantly greater stability compared with acromial correction between 10% and 30% of displacement. However, this difference was not statistically significant when the PABG was added to the severely malaligned condition (Figures 4 [Fig fig5-03635465251362854]-6 and Appendix Tables A1 and A2).

At 30° of flexion, on average, the moderate (99.8 ± 52.9 N) and severe (52.4 ± 48.7 N) malalignment conditions reached 50% of humeral head displacement before 150 N of shear force (Figures 4 [Fig fig5-03635465251362854]-6). In contrast, when a PABG was added, the force limit was reached at approximately 25% of posterior humeral head displacement, and based on the extrapolated data, substantially higher forces would have been required to reach 50% of displacement (moderate: 657.8 ± 448.5 N; severe: 654.5 ± 436.0 N; *P* < .001 and *P* < .001, respectively).

At 60° of flexion, the force limit was reached before 50% of displacement in all conditions (Figure 4). Significantly lower forces were required to translate the humeral head 30% of the glenoid width in both the moderate (65.9 ± 47.1 N) and severe (71.3 ± 49.6 N) malalignment conditions compared with when a PABG was added (moderate: 158.8 ± 77.9 N; severe: 246.8 ± 215.3 N; *P* < .001 and *P* = .021, respectively) (Figures 4 [Fig fig5-03635465251362854]-6).

Compared with moderate malalignment with a PABG, severe malalignment with a PABG provided significantly more stability at 30° of flexion until 15% of displacement. Thereafter, as well as at 60° of flexion, there was no statistically significant difference between both PABG conditions.

### Acromiohumeral Contact Pressure

**Figure 7.**
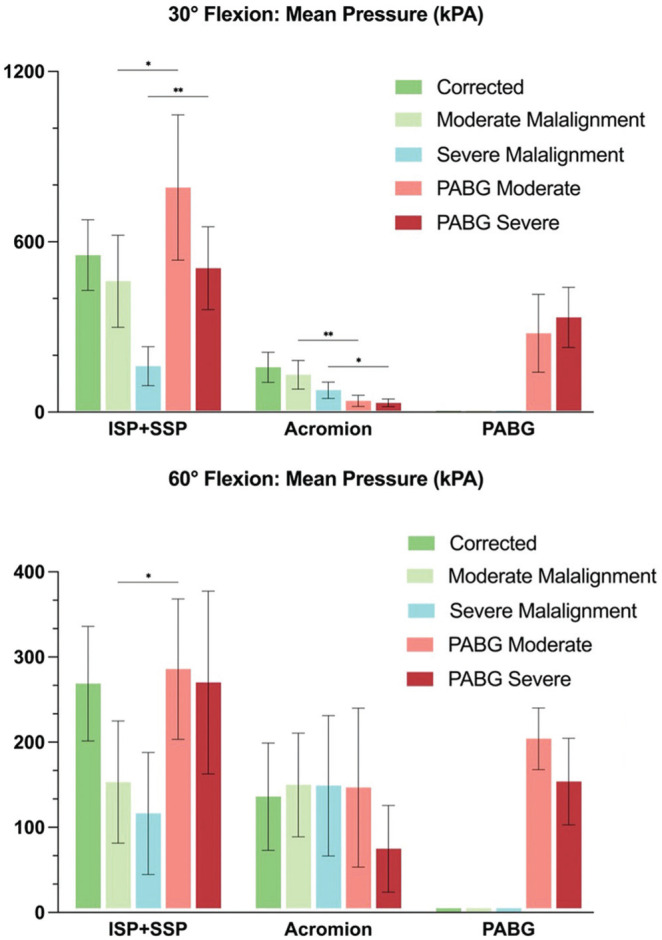
Mean contact pressure per Tekscan sensor location at 30° and 60° of glenohumeral flexion. *P < .05; **P < .01; ***P < .001; ****P < .0001.

At 30° of flexion, mean contact pressure on the rotator cuff was significantly lower for the moderate (460.3 ± 355.9 kPa) and severe (161.9 ± 151.1 kPa) malalignment conditions compared with their PABG-augmented counterparts (moderate: 791.2 ± 562.1 kPa; severe: 507.1 ± 320.9 kPa; *P* = .026 and *P* = .0033, respectively), whereas mean contact pressure was comparable between the corrected acromial alignment and PABG-augmented conditions (Figure 7 and Appendix Tables A3 and A4). Conversely, mean contact pressure on the undersurface of the acromion was significantly higher for the moderate (130.7 ± 110.8 kPa) and severe (77.1 ± 63.3 kPa) malalignment conditions compared with when a PABG was added (moderate: 39.4 ± 42.8 kPa; severe: 32.5 ± 30.0 kPa; *P* = .0031 and *P* = .0494, respectively). Mean contact pressure measured along the PABG was 277.6 ± 301.5 kPa when added to moderate malalignment and 333.3 ± 232.7 kPa when added to severe malalignment (*P* = .9727).

At 60° of flexion, mean contact pressure on the rotator cuff was significantly lower in moderate (153.2 ± 169.9 kPa) but not in severe (116.3 ± 169.9 kPa) malalignment compared with the PABG-augmented conditions (moderate: 285.8 ± 194.9 kPa; severe: 270.1 ± 254.1 kPa; *P* = .0228 and *P* = .0909, respectively) (Figure 7 and Appendix Tables A3 and A4). This lack of statistical significance in the severe malalignment condition may be attributed to high standard variance. Mean contact pressure on the undersurface of the acromion remained comparable throughout all testing conditions. Mean contact pressure measured along the PABG was 204.0 ± 85.9 kPa when added to moderate malalignment and 153.7 ± 120.3 kPa when added to severe malalignment (*P* = .2558).

## Discussion

The most important finding of this study is that a PABG significantly increased resistance to posterior humeral head displacement in shoulders with acromial malalignment and a normal glenoid position and orientation. At both 30° and 60° of glenohumeral flexion, the addition of a PABG to either moderately or severely malaligned acromions enhanced stability after as little as 5% of posterior humeral head displacement, with this stabilizing effect increasing progressively with greater displacement. This finding provides biomechanical evidence supporting Scapinelli’s^29,30^ approach, which reported no recurrence of instability over a 9.6-year follow-up period.

We have previously established that the acromion is high and flat in patients with dynamic and static posterior shoulder instability^1,2,22^ and that it serves as a relevant stabilizer against posterior displacement of the humeral head.^10,14,15^ It seems to provide a mechanical buttress, or “a natural restraint mechanism,” and the increased resistance to posterior humeral head translation observed with PABG augmentation aligns with this biomechanical concept. The current study demonstrates that augmentation with a PABG can effectively compensate for deficient posterolateral acromial coverage by extending this buttress through bone grafting and provides quantitative evidence for this stabilizing effect at least in the presence of a normal glenoid. In shoulders with normal glenoid morphology and an intact posterior capsulolabral complex, we previously found that the force needed to displace the humeral head by 50% of the glenoid width decreased between 23% and 60% in moderate to severe acromial malalignment compared with the native condition.^15^ The current study demonstrates that adding a PABG effectively reversed this instability, limiting displacement to approximately 25% at the 150-N force threshold. The exponential increase in stability with increasing displacement after corrective acromial osteotomy^15^ is mirrored in the current findings by adding a PABG. This suggests that both approaches effectively restore the mechanical buttress function of the acromion.

**Figure 8.**
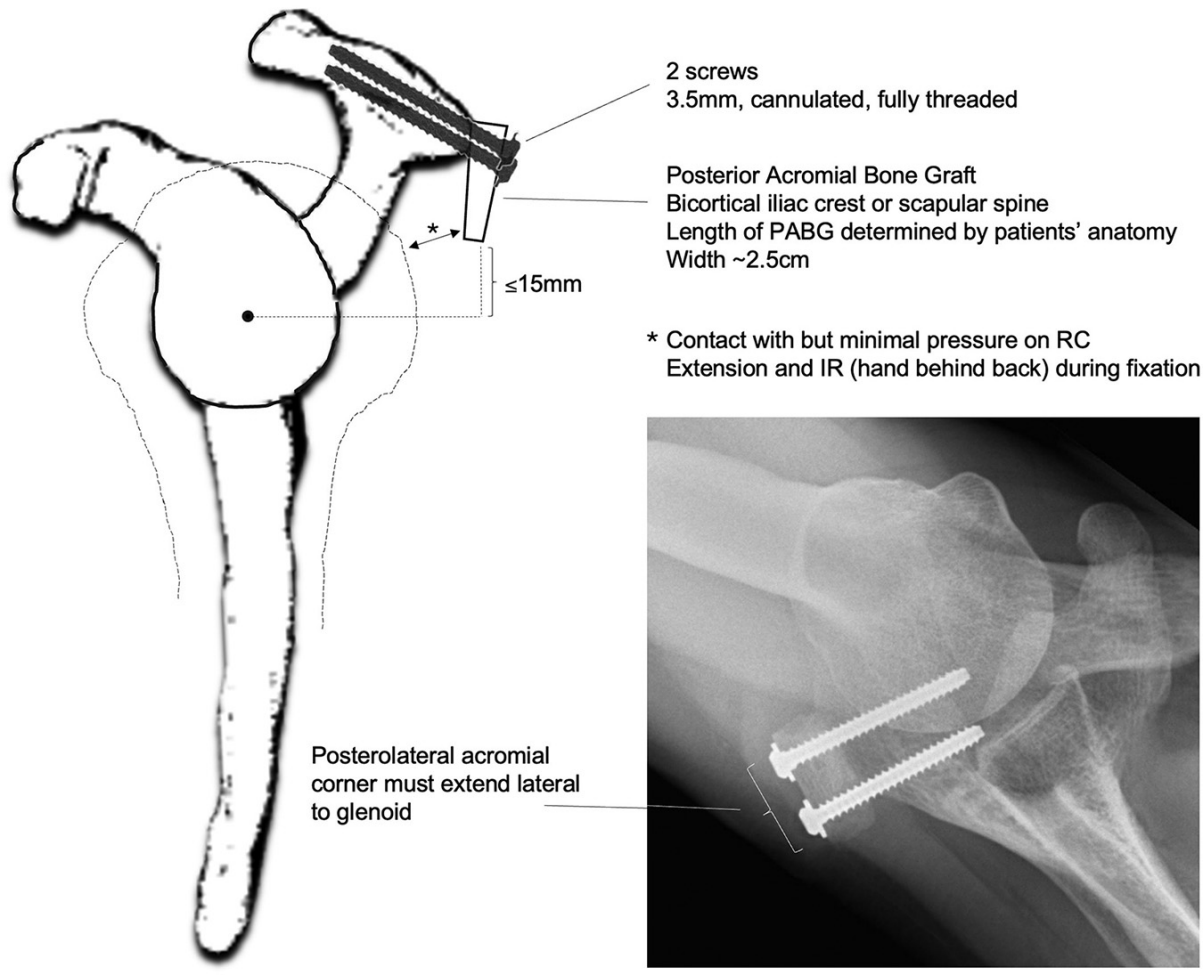
Schematic illustration and radiological image of the posterior acromial bone graft (PABG) technique as currently performed by the authors, highlighting key surgical steps and practical technical pearls.

Another key finding of this study is that the PABG provided comparable or superior stability to surgical acromial correction, depending on the testing position and degree of displacement. At 30° of flexion, the PABG and acromial correction offered similar stability up to 30% of displacement, beyond which the PABG actually provided significantly greater resistance. At a theoretical 50% of displacement, it would require substantially higher forces to dislocate a PABG-augmented shoulder compared with one with corrected acromial alignment. This finding has significant clinical implications, as it suggests that a PABG, a technically simpler procedure than 3D corrective osteotomy, may achieve, at worst, equivalent and, at best, superior biomechanical stability. We attribute this finding to the chosen size and positioning of the bone graft, which augmented the posterolateral corner of the acromion more distinctly than would be possible with reorienting corrective osteotomy. It essentially represents an overcorrection of the acromion. Statements about the ideal or necessary size and positioning (mediolateral or the angle to the acromion) of the PABG cannot be made with our data; further biomechanical or clinical studies are needed. However, it seems important to note that based on our preliminary clinical experience, a PABG cannot always be used effectively. In cases with a dysplastic acromion, the posterolateral corner is often difficult to define and may lie medial to the humeral head. Because the PABG aims to provide a mechanical buttress to the humeral head, it needs to be positioned laterally enough. For such cases, corrective acromial osteotomy, sometimes combined with a PABG, might be necessary. With the current knowledge on acromial anatomy, we believe that meticulous preoperative (3D) planning is essential, as the goal is to restore the physiological PAH and posterolateral humeral head coverage. This frequently results in a graft size of approximately 2.5 × 2.5 cm. We commonly use bicortical iliac crest autografts, as they allow for more reliable harvesting of the planned graft dimensions and avoid weakening the scapular spine. In our view, the choice of graft donor site is unlikely to affect the biomechanical principle or clinical effectiveness of the PABG. Nevertheless, as Scapinelli^30^ was the first to propose this technique, we believe that it is appropriate to reference his original concept and to acknowledge his contribution, even though both the biomechanical and clinical implementation have been adapted. Intraoperatively, to avoid overcorrection, we extend and internally rotate the arm (positioning the patient's hand on his or her back) to ensure minimal pressure on the rotator cuff. To fix the PABG, we use cannulated 3.5-mm fully threaded screws with a washer (Figure 8).

The stability provided by the PABG appears to be a continuum that varies with the degree of malalignment, similar to previous observations regarding acromial morphology itself. This raises the possibility that a PABG might allow surgeons to compensate for minor malalignment of the glenoid without requiring additional glenoid procedures. Furthermore, a PABG could be an option in patients with a very dysplastic short acromion in which the native acromion itself does not provide sufficient posterolateral coverage, even after corrective osteotomy: either as an alternative to or in addition to osteotomy. Overall, the PABG performed similarly across both degrees of malalignment without statistically significant differences. However, the PABG in the severe malalignment condition tended to provide greater stability. This may be attributed to the fact that in the severe malalignment condition, the posterolateral acromion was positioned slightly more anteriorly, resulting in the posteriorly placed graft also being situated slightly more anteriorly.

The alterations in acromiohumeral contact patterns observed in this study provide insight into the mechanism by which the PABG enhances stability. It restored contact pressure on the rotator cuff compared with the other conditions and absorbed a significant amount of pressure while decreasing pressure on the acromion's undersurface, particularly at 30° of flexion. This redistribution suggests that a PABG functions by (1) creating a direct mechanical block to posterior translation and (2) normalizing the load distribution across the rotator cuff. Consistent pressure measured along the PABG in both the moderate and severe malalignment conditions indicates that the graft provided a reliable contact surface, regardless of the underlying degree of malalignment. This finding supports the potential versatility of this technique across a spectrum of anatomic variants. It could be argued that enhanced coverage of the humeral head and the resultant increase in acromiohumeral contact pressure may lead to atypical loading on the humeral head, potentially triggering osteoarthritis through the introduction of abnormal movement patterns, particularly the natural dorsocranial shift of the center of rotation during active movements. We contend that the protective layer of the infraspinatus muscle between the humeral head and the acromion may offset any problems related to increased cartilage contact pressure on the humeral head. However, further studies are required to verify this and to ensure that there is no significant impingement of the posterosuperior rotator cuff. Additionally, more research is needed to investigate possible alterations in alignment, joint kinematics, and contact pressure in the acromioclavicular and sternoclavicular joints.

Understanding the risk factors for primary and recurrent posterior shoulder instability is crucial, given that posterior shoulder instability accounts for up to 18% of primary glenohumeral instability events and up to 24% of surgical stabilization procedures, with failure rates for commonly used surgical treatment options between 35% and 73% at long-term follow-up.^3,5,8,12,13,16,28,33,34,37^ These high failure rates might be explained by the fact that the acromion's morphology has historically not been recognized as a relevant stabilizing element of the shoulder. Given the high failure rates of traditional approaches, a PABG offers an alternative that addresses a different anatomic aspect of posterior instability and targets a structural deficiency that remains unaddressed by conventional surgical approaches focused on the glenoid or capsulolabral complex. While the relevance of the posterior capsulolabral complex is debatable (Is it the cause or a consequence of instability?), in combined abnormalities, a PABG might serve as a valuable adjunct to traditional soft tissue repair. Testa et al^32^ conducted a cadaveric biomechanical study and showed that adding a posterior acromial bone block to a native specimen produced higher resistance forces than labral repair alone. However, both scenarios were comparable with the resistance forces produced by the intact specimens likely because the acromial morphology of the intact specimens was normal.

Gerber et al^10^ described a 3D-planned acromial and glenoid osteotomy technique (SCOPE procedure) to restore scapular anatomy in cases of static, dynamic, or mixed posterior instability. In this case report of a patient with static posterior instability, the goal was to prevent early osteoarthritis, and at 2 years, humeral head recentering with good clinical outcomes was reported. Similar principles may apply to the PABG, which could offer a less invasive alternative with comparable benefits in selected patients with static posterior instability and early degenerative changes.

### Limitations

This study has various limitations. The sample size was based on an a priori power analysis from a small pilot study, which makes the effect size estimates susceptible to sampling variability and potential inaccuracy. Because the power analysis was based solely on force-displacement data, statistical comparisons involving acromiohumeral contact pressure may be underpowered and should be interpreted with appropriate caution. However, most comparisons yielded statistically significant results, and the relevance of the power analysis is therefore limited in this context. Static and dynamic stabilizing factors cannot be fully replicated in a cadaveric model. Bone and soft tissue quality of the specimens as well as the testing setup may differ from in vivo conditions, and the testing sequence may have had a cumulative effect on tissue quality. In clinical situations, acromial morphology may be the only pathological feature of a scapula, but it often is not. The results should be interpreted in light of the fact that we consciously excluded posterior capsulolabral lesions, abnormalities in glenoid version and inclination, and glenoid bone loss, as it was the study's purpose to examine the relevance of acromial variants on the risk of developing posterior instability. The relevance of the excluded factors and their effect on posterior translation have already been biomechanically validated and pertain particularly to recurrent instability. Nonetheless, the study further confirms that the previously neglected acromion has a clear role in the stability of the glenohumeral joint. A testing sequence was chosen in which the most malaligned acromial morphology was tested before all other surgical interventions to ensure the most stable soft tissue situation and allow for more soft tissue laxity in the corrected acromion condition to minimize this bias. While designed to replicate clinical examination maneuvers, our testing protocols represent simplified conditions compared with the complex forces experienced during athletic activities or activities of daily living. All testing was performed on specimens with intact capsulolabral structures, whereas clinical posterior instability often involves concomitant abnormalities that might alter the effect of a PABG. This study assessed only immediate mechanical effects without accounting for biological integration, remodeling, or potential long-term complications (ie, altered pressure on the rotator cuff). Only one standard size and configuration of the PABG was tested; various dimensions, shapes, or fixation methods might yield different biomechanical effects. The Tekscan sensor foils used were small and did not cover the entire rotator cuff or acromial undersurface, potentially underestimating contact pressure, particularly in the corrected acromion condition. Additionally, planar Tekscan sensors are not perfectly designed for assessing pressure between curved surfaces such as the humeral head and the undersurface of the acromion, which can lead to sensor bending. To reduce this bending issue, phosphate-buffered saline was used between sensors before each test. Furthermore, to avoid damage to the sensors, they were replaced after the complete testing cycle of each specimen. The use of polynomial curve fitting and extrapolation to estimate forces beyond the testing limit (150 N) introduces a potential source of error. While this approach allowed for statistical comparisons across scenarios, the extrapolated values should be interpreted with caution. These limitations suggest opportunities for future research, including clinical studies, to validate these biomechanical findings.

## Conclusion

The study provides quantitative evidence showing that a PABG significantly enhanced resistance to displacement and compensated for deficient posterolateral acromial coverage by extending the natural mechanical buttress. Experimentally, the PABG provided comparable or superior stability to that after surgical acromial reorientation while representing a technically simpler and potentially less invasive approach.

## Supplemental Material

sj-pdf-1-ajs-10.1177_03635465251362854 – Supplemental material for Posterior Shoulder Stability Can Be Restored by Posterior Acromial Bone Grafting (Scapinelli) in a Cadaveric Biomechanical Model With Normal Glenoid Anatomy Supplemental material, sj-pdf-1-ajs-10.1177_03635465251362854 for Posterior Shoulder Stability Can Be Restored by Posterior Acromial Bone Grafting (Scapinelli) in a Cadaveric Biomechanical Model With Normal Glenoid Anatomy by Bettina Hochreiter, Nhi Nguyen, Anna-Katharina Calek, Bastian Sigrist, David C. Ackland, Lukas Ernstbrunner, Eugene T. Ek and Christian Gerber in The American Journal of Sports Medicine
