# Supplementary material for: Posterior Shoulder Stability Can Be Restored by Posterior Acromial Bone Grafting (Scapinelli) in a Cadaveric Biomechanical Model With Normal Glenoid Anatomy
Source: Am J Sports Med. 2025 Aug 18;53(11):2684–94. doi: 10.1177/03635465251362854 (PMC12381383; doi:10.1177/03635465251362854)
Supplement: sj-pdf-1-ajs-10.1177_03635465251362854 – Supplemental material for Posterior Shoulder Stability Can Be Restored by Posterior Acromial Bone Grafting (Scapinelli) in a Cadaveric Biomechanical Model With Normal Glenoid Anatomy [file sj-pdf-1-ajs-10.1177_03635465251362854.pdf]

APPENDIX

Table 1. Force data (mean ±SD) between 5; 50% of posterior humeral head displacement in 5% increments in 30° and 60° of glenohumeral flexion. Please see figures 5 and 6 in the manuscript for comparisons between conditions and p-values.

	Force (N)									
**Displacement (%)**	**Corrected**		**PABG Moderate**		**Moderate malalignment**		**PABG Severe**		**Severe Malalignment**	
**30° of flexion**	*Mean*	*SD*	*Mean*	*SD*	*Mean*	*SD*	*Mean*	*SD*	*Mean*	*SD*
5	13.5	12.0	9.8	8.8	4.5	3.2	18.6	17.7	4.3	2.3
10	25.0	19.8	23.4	20.9	8.4	6.5	47.8	40.1	7.6	4.2
15	42.1	22.9	45.5	34.4	13.9	8.7	74.8	53.9	11.2	6.0
20	65.5	26.8	77.9	50.0	19.9	9.1	119.6	83.0	14.5	7.5
25	90.8	33.9	124.6	69.7	28.1	11.4	175.4	121.7	17.8	9.6
30	119.4	43.9	185.0	101.1	37.1	14.9	243.0	170.0	21.2	12.5
35	156.0	61.2	266.6	147.1	47.3	21.1	323.7	225.7	25.8	17.8
40	194.8	85.0	370.6	216.8	60.8	29.5	417.7	290.5	31.9	25.7
45	239.6	113.6	500.0	315.6	78.4	40.7	527.3	361.1	40.2	35.1
50	288.3	149.0	657.8	448.5	99.8	52.9	654.5	436.0	52.4	48.7
**60° of flexion**										
5	13.7	10.9	9.8	6.7	5.6	3.8	12.5	12.5	5.9	4.6
10	36.1	25.3	19.7	13.0	12.4	8.4	32.1	34.8	12.8	9.6
15	67.0	42.1	35.9	24.7	21.2	14.8	63.3	68.4	21.6	15.1
20	110.5	66.7	62.5	41.2	32.3	23.9	105.9	107.8	33.7	22.9
25	166.3	95.1	101.8	59.0	46.4	34.6	166.7	157.0	49.8	35.7
30	234.3	129.1	158.8	77.9	65.9	47.1	246.8	215.3	71.3	49.6
35	316.3	168.5	242.9	100.9	92.4	61.0	350.0	285.0	101.1	68.7
40	413.3	213.3	347.1	128.2	126.4	75.9	477.6	372.7	136.6	97.7
45	526.2	264.6	490.6	177.4	170.1	91.2	633.9	483.2	182.6	136.7
50	655.8	324.1	657.6	234.5	223.9	108.2	829.3	619.4	238.3	190.0

Table 2. Force data (mean differences and 95% confidence intervals) between 10% and 50% of posterior humeral head displacement in 10% increments in 30° and 60° of glenohumeral flexion.

**Δ Force (N)**	**Displacement (%)**									
	**10%**		**20%**		**30%**		**40%**		**50%**	
**30° of flexion**	**Δ** *Mean*	*95% CI*	**Δ** *Mean*	*95% CI*	**Δ** *Mean*	*95% CI*	**Δ** *Mean*	*95% CI*	**Δ** *Mean*	*95% CI*
Moderate Malalignment – PABG Moderate	-15.1	-26.2; -3.9	-58.0	-84.3; -31.8	-147.8	-199.7; -96.0	-309.8	-417.8; -201.9	-558.0	-780.4; -335.6
Corrected – PABG-Moderate	1.5	-14.9; 18.1	-12.4	-40.2; 15.3	-65.6	-125.2; -5.9	-175.8	-300.4; -51.2	-369.5	-618.2; -120.9
Severe Malalignment – PABG Severe	-40.2	-59.3; -21.2	-105.1	-145.6; -64.6	-221.7	-306.6; -136.9	-385.8	-532.8; -238.9	-602.1	-824.5; -379.6
Corrected – PABG Severe	-22.8	-44.5; -1.2	-54.1	-101.7; -6.5	-123.6	-215.0; -32.2	-222.9	-371.5; -74.3	-366.2	-582.7; -149.7
**60° of flexion**										
Moderate Malalignment – PABG Moderate	-7.3	-12.4; -2.3	-30.3	-45.5; -15.0	-92.9	-129.3; -56.5	-220.8	-292.8; -148.7	-433.7	-571.4; -295.9
Corrected – PABG-Moderate	16.4	5.8; 27.1	47.9	26.8; 69.1	75.5	30.7; 120.3	66.2	-22.6; 154.9	-1.8	-169.9; 166.4
Severe Malalignment – PABG Severe	-19.3	-35.7; -3.0	-72.3	-124.5; -20.0	-175.4	-288.5; -62.4	-340.9	-551.2; -130.7	-591.0	-957.5; -224.5
Corrected – PABG Severe	4.0	-12.7; 20.7	4.5	-40.9; 49.9	-12.5	-104.0; 79.0	-64.29	-226.4; 97.9	-173.5	-445.5; 98.6

Table 3. Average contact pressure (mean ±SD) in 30° and 60° of glenohumeral flexion. Please see figure 7 in the manuscript for comparisons between conditions and p-values.

	Average Contact Pressure (kPA)									
**Location**	**Corrected**		**PABG Moderate**		**Moderate malalignment**		**PABG Severe**		**Severe Malalignment**	
**30° of flexion**	*Mean*	*SD*	*Mean*	*SD*	*Mean*	*SD*	*Mean*	*SD*	*Mean*	*SD*
**ISP+SSP**	552.9	273.6	791.2	562.1	460.3	355.9	507.1	320.9	161.9	151.1
**Acromion**	158.0	116.0	39.4	42.8	130.7	110.8	32.5	30.0	77.1	63.3
**PABG**			277.6	301.5			333.3	232.7		
**60° of flexion**										
**ISP+SSP**	268.7	159.4	285.8	194.9	153.2	169.9	270.1	254.1	116.3	169.9
**Acromion**	136.0	149.4	146.7	220.9	149.8	144.1	74.8	120.5	148.9	195.1
**PABG**			204.0	85.9			153.7	120.3		

Table 4. Average contact pressure (mean differences and 95% confidence intervals) in 30° and 60° of glenohumeral flexion.

Δ Average Contact Pressure (kPA)	Location			
	ISP+SSP		Acromion	
**30° of flexion**	Δ *Mean*	*95% CI*	Δ *Mean*	*95% CI*
Corrected – PABG Moderate	-238.3	-697.4; 220.9	118.6	55.1; 182.2
Corrected – PABG Severe	45.8	-302.0; 393.7	125.5	42.9; 208.0
Moderate – PABG Moderate	-330.9	-630.5; -31.3	91.4	27.2; 155.5
Severe – PABG Severe	-345.2	-589.3; -101.2	44.5	0.1; 89.0
**60° of flexion**				
Corrected – PABG Moderate	-17.1	-140.8; 106.6	-10.7	-137.7; 116.3
Corrected – PABG Severe	-1.3	-145.8; 143.1	61.2	15.0; 107.5
Moderate – PABG Moderate	-132.6	-250.9; -14.4	3.1	-121.4; 127.7
Severe – PABG Severe	-153.8	-324.4; 16.8	74.1	-46.8; 194.9
